# The Au–CeO_2_ Nanozyme with Enhanced Peroxidase-like Activity for Highly Sensitive Ratiometric Colorimetric Detection of Nitrite

**DOI:** 10.3390/foods15213881

**Published:** 2026-10-29

**Authors:** Luyu Chen, Wenting Lu, Chunyan Sun, Yingrui Zhang, Jicai Cai, Jiaqi Chen, Linna Du, Jiajun Hu, Jing Qin

**Affiliations:** 1 College of Advanced Materials Engineering, Jiaxing Nanhu University, Jiaxing 314001, China; 19817626673@163.com (L.C.); 18168842670@163.com (W.L.); 13663780247@163.com (C.S.); 17357350817@163.com (Y.Z.); 15236911235@163.com (J.C.); cherry_0412@foxmail.com (J.C.); linnadu1983@jxnhu.edu.cn (L.D.); 2 Zhejiang Huayou Cobalt Co., Ltd., Jiaxing 314599, China; hujiajun@huayou.com; 3 Green Food Technology Innovation Laboratory, Jiaxing Nanhu University-Zhejiang Wufangzhai Industrial Co., Ltd., Jiaxing 314001, China

**Keywords:** Au–CeO_2_ nanozyme, peroxidase-like activity, ratiometric colorimetric assay, nitrite detection, diazotization reaction

## Abstract

Nitrite (NO_2_^−^) is a common food additive but it poses serious health risks due to its potential to form carcinogenic N-nitrosamines. Rapid and sensitive detection of nitrite in food matrices remains a critical need. Herein, we report a ratiometric assay based on Au-loaded mesoporous CeO_2_ (Au–CeO_2_) nanozymes with enhanced peroxidase-like activity for highly sensitive nitrite detection. The Au–CeO_2_ nanozyme was synthesized via a facile thermal decomposition and reduction method. Au–CeO_2_ nanozyme exhibited a higher Ce^3+^/Ce^4+^ ratio (from 25:75 to 31:69) and increased oxygen vacancy density (from 22.4% to 31.2%) compared to pristine CeO_2_. Meanwhile, the mesoporous structure effectively enhances the interaction between catalytic substrates and the active sites of nanozymes. The mesoporous structure is favorable for substrate accessibility to the active sites, which may contribute to the enhanced peroxidase-like catalytic performance. The nanozyme efficiently catalyzed the oxidation of 3,3′,5,5′-tetramethylbenzidine (TMB) to oxTMB, producing a strong absorbance at 652 nm. Upon the addition of nitrite, diazotization of oxTMB occurred, leading to a decreased signal at 652 nm and a new absorbance peak at 445 nm. This ratiometric response (A_652_/A_445_) enabled sensitive detection of NO_2_^−^ in the range of 1–200 μM with a detection limit of 0.43 μM. The ratiometric colorimetric assay demonstrated excellent selectivity and was successfully applied to real food samples with a satisfactory recovery (95.4–106.6%, RSD < 2.73%). The ratiometric colorimetric method provides a novel analytical strategy for environmental and food safety monitoring, offering high sensitivity, high accuracy, and excellent reliability.

## 1. Introduction

Nitrite (NO_2_^−^), which serves as a food additive, is extensively found in drinking water and food [1]. However, when NO_2_^−^ reacts with amines, amides, or proteins, it has the potential to generate highly carcinogenic *N*-nitrosamine molecules. Prolonged excessive intake of nitrite poses severe health risks to humans [2,3]. Therefore, the development of a simple and rapid method for detecting nitrite in food is essential. Currently, various methods for NO_2_^−^ detection have been developed, including electrochemical [4], fluorescent [5,6,7], and gas chromatographic [8] techniques. However, these traditional methods often face drawbacks such as the need for large-scale and expensive instruments, specialized operational expertise, complex procedures, and lengthy analysis times. In contrast, colorimetric analysis based on colored compounds has gained increasing attention, owing to their affordability, operational simplicity, capability for rapid visual detection, and potential for on-site applications [9,10,11,12]. Nevertheless, constructing an efficient catalyst remains a critical step in enhancing the performance of colorimetric detection.

Importantly, nanozymes have emerged as versatile catalysts for chromogenic reactions in numerous colorimetric biosensing platforms, enabling signal amplification to enhance detection sensitivity [13,14,15,16]. Among them, CeO_2_ nanozyme has demonstrated multifunctional enzymatic behaviors including peroxidase (POD)-like activities. This is primarily attributed to their rich oxygen vacancy content and reversible Ce^3+^/Ce^4+^ redox transitions [17,18,19]. To improve catalytic efficiency by facilitating electron transfer and lowering activation energy, researchers have adopted diverse structural engineering strategies for metal oxide nanozymes. These include precise control of cerium valence distribution (Ce^3+^/Ce^4+^ ratio) [20,21], heteroatom doping with transition metals [22,23,24], surface functionalization via ligand grafting [25,26], and deliberate modulation of crystalline phase and lattice symmetry [27]. The deposition of Noble metals on metal oxide nanozymes has been shown to induce strong metal-support interactions (SMSI) that facilitate charge redistribution and enhance reactive oxygen species (ROS) generation, thereby amplifying catalytic activity [28,29]. For example, metal deposition can modulate the electronic structure, increase oxygen vacancy density, and promote interfacial electron transfer [30].

Furthermore, mesoporous structures provide a higher specific surface area and are capable of accommodating more active sites, thereby effectively improving the catalytic efficiency of nanozymes. The nanoscale confinement effect within mesopores results in the local enrichment of substrate molecules due to the restricted translational degrees of freedom, which significantly increases the collision frequency between reactants and active sites [31,32]. Mesoporous confinement is also capable of modulating the d-band center of metal active sites, optimizing the adsorption free energy of key intermediates (such as *OH and *OOH) [33,34]. Consequently, mesoporous structures lower the activation energy barrier of the rate-determining step and enhance the catalytic efficiency of nanozymes [35,36]. Moreover, the mesoporous channels are expected to facilitate the diffusion of reactants and products toward the active sites, which is beneficial for improving the catalytic efficiency.

**Figure 1:**
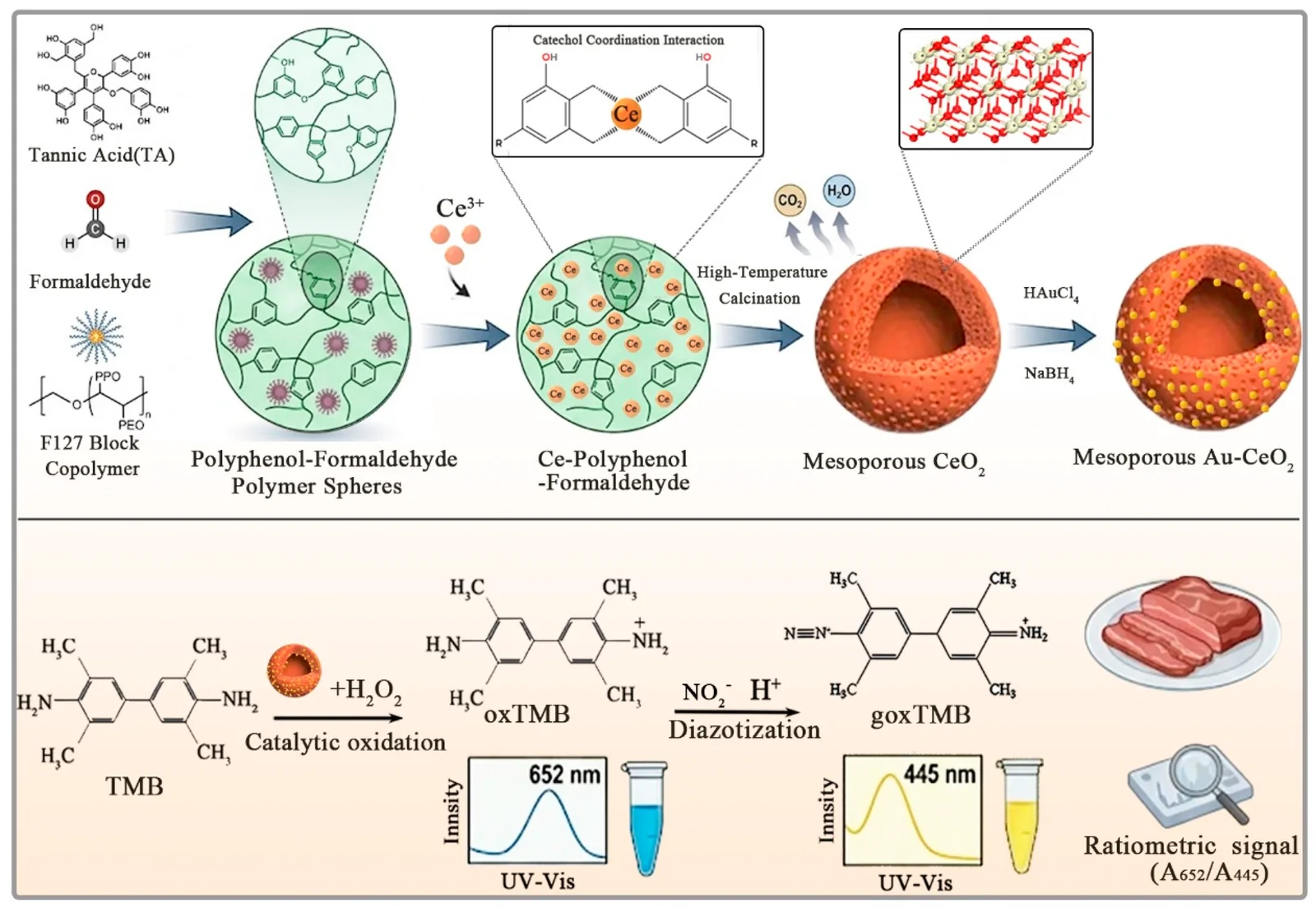
Schematic representation of the synthesis of Au–CeO_2_ nanozyme and detection of NO_2_^−^.

In this study, a straightforward and practical approach was developed to fabricate Au-decorated CeO_2_ (Au–CeO_2_) as a nanozyme platform for highly sensitive ratiometric nitrite sensing (Figure 1). The incorporation of Au nanoparticles into the CeO_2_ matrix significantly boosted its intrinsic peroxidase-mimicking capability—attributed to an increased concentration of oxygen vacancies and a higher Ce^3+^/Ce^4+^ ratio relative to pristine CeO_2_. In the presence of hydrogen peroxide, Au–CeO_2_ efficiently catalyzes the oxidation of TMB to its blue-colored oxTMB, which exhibits a strong absorbance peak at 652 nm. Crucially, NO_2_^−^ selectively react with oxTMB via diazotization, yielding a yellow diazotized derivative that displays enhanced absorption at 445 nm while concurrently diminishing the signal at 652 nm (Figure 1). Accordingly, a robust ratiometric colorimetric detection system was constructed, leveraging this dual-wavelength response that is rooted in both the enzyme-mimetic behavior of Au–CeO_2_ and the specific chemical interaction between NO_2_^−^ and oxTMB. This method delivered high sensitivity, excellent selectivity, reproducibility, and applicability in complex food samples, highlighting its promising utility for ensuring food safety.

## 2. Materials and Methods

### 2.1. Synthesis of Mesoporous CeO_2_ and Au–CeO_2_ Nanozyme

Mesoporous CeO_2_ nanozymes were prepared through thermal calcination of a cerium–tannic acid (CeTA) coordination polymer precursor. Briefly, Pluronic F127 (0.1 g) was first dispersed in a homogeneous solution comprising deionized water, ethanol, and aqueous ammonia. After homogenization for 1 h, an 8 mL TA solution was introduced. 5 min later, 3.8 mL of CH_2_O solution was added dropwise. The resulting mixture was continuously stirred for 12 h to facilitate polymerization. Subsequently, 2 mL of cerium nitrate hexahydrate solution (35 mg/mL) was incorporated, and the system was aged for another 12 h. The suspension was then subjected to hydrothermal treatment at 100 °C for 12 h. Solid products were collected by centrifugation, washed thoroughly, and dried under ambient conditions. Finally, the obtained polymeric solid was calcined in air at 400 °C for 4 h to generate crystalline mesoporous CeO_2_ nanozymes.

Synthesized CeO_2_ nanozyme (50 mg) and HAuCl_4_ aqueous solution (0.1 M) were dispersed in 50 mL of water. Then, 2 mL of freshly prepared NaBH_4_ solution was added as a reducing agent, and the mixture was stirred until the color change stabilized, indicating the complete reduction and deposition of Au. The resulting Au–CeO_2_ nanozyme was collected by centrifugation and vacuum drying. To obtain optimal activity, different mass ratios of HAuCl_4_ and CeO_2_ nanozyme (1%, 2%, 3% and 5%) were used to prepare Au–CeO_2_ nanozyme. They were denoted Au(x)–CeO_2_ (x = 1%, 2%, 3% and 5%).

### 2.2. Validation of the Peroxidase-like Activity of Au–CeO_2_ Nanozyme

The peroxidase-like performance of the Au–CeO_2_ nanozyme was evaluated via the chromogenic assay between TMB and H_2_O_2_. Specifically, 10 μL of Au–CeO_2_ nanozyme (0.5 mg/mL), 50 μL of TMB (0.5 mM), and 50 μL of H_2_O_2_ (5 mM) were sequentially introduced into 890 μL of 10 mM NaAc–HAc buffer solution (pH 4.0). The mixture was then allowed to react for 20 min, and the change in absorbance of A_652_ was measured.

By systematically adjusting the pH (3–7) and temperature (0–60 °C) of the reaction system, we investigated the optimal conditions for the catalytic activity of Au–CeO_2_ nanozyme. The catalytic activity observed under these optimal reaction conditions is defined as 100%, while all other levels of catalytic activity are expressed as a percentage relative to this maximum value.

By using 5,5-dimethyl-1-pyrroline-N-oxide (DMPO) and 2,2,6,6-tetramethylpiperidin-1-oxyl (TEMP) to capture the reactive oxygen species in peroxidase-like activity, EPR measurements were conducted. Specifically, 30 μL of Au–CeO_2_ nanozymes (1 mg/mL), 50 mM DMPO and TEMP, and 0.1 M H_2_O_2_ were mixed in an acetic acid-sodium acetate buffer solution with a pH of 4 and a concentration of 10 mM.

Additionally, the Michaelis–Menten constant (*K*_M_) and maximum velocity (*V*_max_) were determined using the Michaelis–Menten equation.

(1) $$v=\frac{V_{\max}\cdot \left[S\right]}{K_{M}+\left[S\right]}$$

(2) $$\frac{1}{V}=\frac{K_{M}}{V_{\max}}\cdot \frac{1}{\left[S\right]}+\frac{1}{V_{\max}}$$

### 2.3. Ratiometric Colorimetric Sensing of NO_2_^−^

A ratiometric colorimetric sensing assay was developed through Au–CeO_2_ nanozyme-catalyzed oxidation of TMB and NO_2_^−^ -triggered diazotization. In brief, 10 μL of Au–CeO_2_ nanozyme (0.50 mg/mL), 50 μL of TMB (0.5 mM), and 50 μL of H_2_O_2_ (5 mM) were introduced into 890 μL of NaAc–HAc buffer solution (10 mM, pH 4.0). The mixture was then allowed to react for 20 min, resulting in the formation of oxTMB. Subsequently, 10 μL of NO_2_^−^ standard solutions (1–200 μM) was added, and the mixture was allowed to react for another 15 min. The ratio of absorbance at 652 nm to that at 445 nm (A_652_/A_445_) served as the quantitative ratiometric output, facilitating sensitive and linear detection of NO_2_^−^ across the specified concentration range.

Meanwhile, the specificity of this method was evaluated through anti-interference experiments. 10 μL of Au–CeO_2_ nanozyme (0.50 mg/mL), 50 μL of TMB (0.5 mM), and 50 μL of H_2_O_2_ (5 mM) were added to 890 μL of NaAc–HAc buffer solution (10 mM, pH 4.0). After 20 min of reaction, 50 μM of various interfering substances (K^+^, NH_4_^+^, Mg^2+^, Mn^2+^, Cu^2+^, NO_3_^−^, Cl^−^, SO_4_^2−^, PO_4_^3−^, Gly (glycine), His (histidine), Tyr (tyrosine), Glu (glutamic acid) and NO_2_^−^ were introduced into the reaction mixture. Following a 15 min incubation period, UV–vis absorption spectra were acquired.

### 2.4. Evaluation of the Ratio Detection Mode and Single Wavelength Detection Mode

To evaluate whether the ratiometric detection mode offers an analytical advantage over single-wavelength measurements, a controlled recovery experiment was performed using a colored interferent (crystal violet) to simulate proportional background absorbance at both 652 nm and 445 nm. 

In a typical procedure, 10 μL of Au–CeO_2_ nanozyme (0.5 mg/mL), 50 μL of TMB (0.5 mM), and 50 μL of H_2_O_2_ (5 mM) were mixed in 890 μL of NaAc–HAc buffer (10 mM, pH 4.0), and the mixture was allowed to react for 20 min to form oxTMB. Subsequently, 10 μL of NO_2_^−^ standard solution was added to give a final concentration of 5 μM, and the mixture was reacted for another 15 min. By adding crystal violet, a colored background was introduced. The low, medium and high levels of background represent the concentrations of crystal violet as 0.05, 0.1 and 0.5 mg/mL respectively. For each background level, a corresponding background blank was prepared identically but without NO_2_^−^. UV–vis spectra were recorded from 350 to 700 nm, and the absorbance values at 652 nm and 445 nm were read from the same spectra.

### 2.5. Detection of NO_2_^−^ in Real Samples

Fermented vegetables and salted duck eggs were acquired from supermarket and subjected to sample preparation prior to analysis. For each test, a 5 g portion was homogenized in a 50 g/L borax solution using a high-speed blender to yield a homogeneous slurry. This slurry was then transferred to ultrapure water and incubated at 70 °C for 15 min. After heating, protein precipitation was achieved by adding K_4_[Fe(CN)_6_] (106 g/L) and Zn(Ac)_2_ (220 g/L). The resulting mixture was diluted with ultrapure water to ensure consistency and clarity. Finally, NO_2_^−^ standard solutions of defined concentrations were added to the processed samples for quantitative determination.

To minimize interference from intrinsic sample color, the following measures were adopted. The extracts were diluted with ultrapure water to reduce the absolute concentration of colored pigments. A matrix blank (the same extract processed identically but without TMB/H_2_O_2_) was measured at both wavelengths and subtracted from the corresponding sample readings. 

## 3. Results

### 3.1. Synthesis and Characterization of Au–CeO_2_ Nanozyme

**Figure 2:**
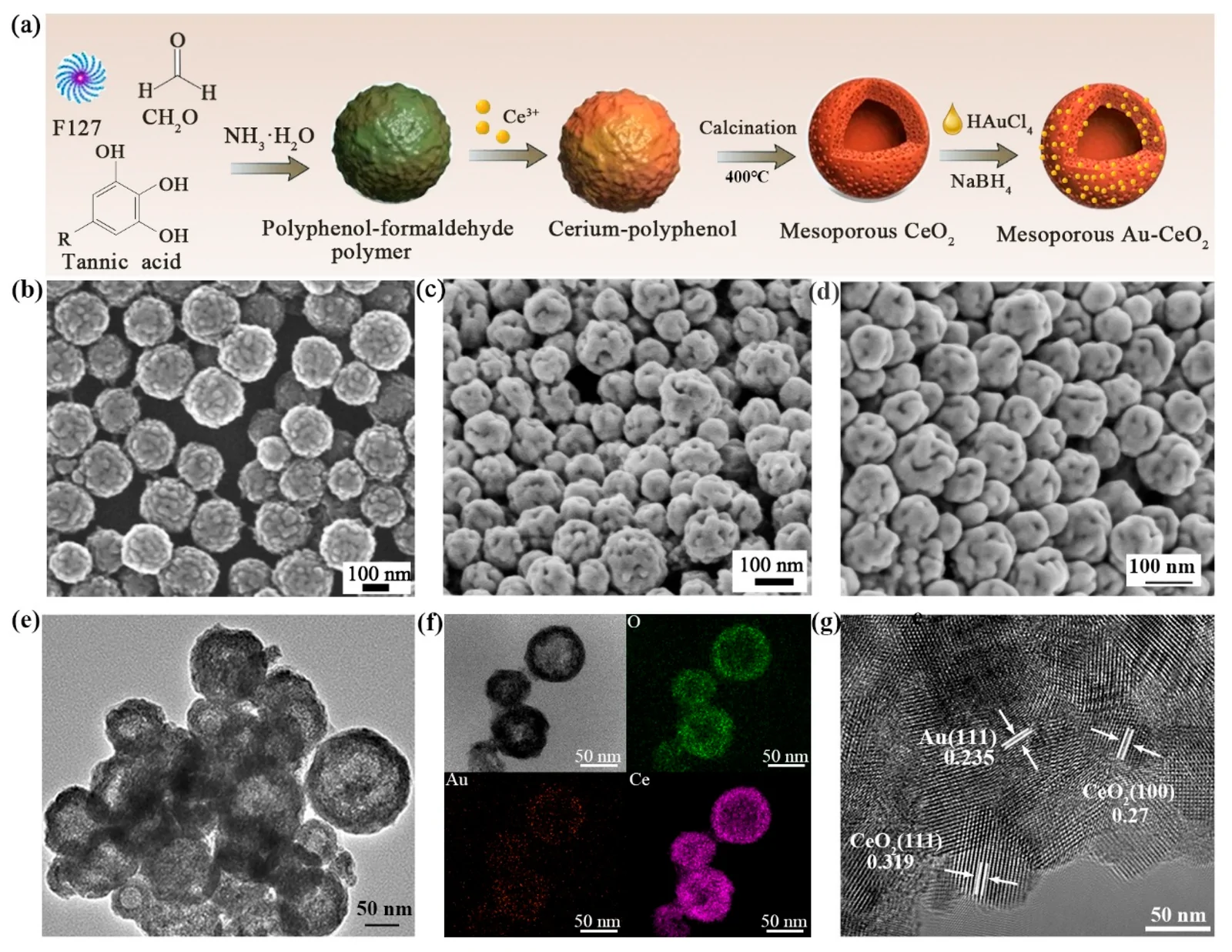
(**a**) Schematic representation of the synthesis of Au–CeO_2_ nanozyme. (**b**) SEM images of Ce-TA polymers. (**c**) SEM images of CeO_2_. (**d**) SEM and (**e**) TEM images of Au–CeO_2_ nanozyme. (**f**) Element mapping of Au–CeO_2_ nanozyme. (**g**) HR-TEM images of Au–CeO_2_ nanozyme.

The mesoporous Au–CeO_2_ nanozyme was constructed through the following steps. Firstly, cerium-polyphenol-formaldehyde (Ce-TA) polymers were synthesized using a modified sol–gel process with tannic acid as the ligand, Ce^3+^ as the metal source, and formaldehyde as the crosslinker (Figure 2a). Secondly, mesoporous CeO_2_ nanozyme was prepared by a direct thermal decomposition of Ce-TA polymers at 400 °C for 4 h. During the calcination process, the decomposition of the organic framework led to significant shrinkage, resulting in CeO_2_ nanozymes with a mesoporous structure (Figure 2a). Thirdly, after the chemical reduction of HAuCl_4_ by NaBH_4_, Au nanoparticles were in situ anchored on the surface of the CeO_2_ nanozyme, resulting in the final product, Au–CeO_2_ nanozyme (Figure 2a).

The scanning electron microscope (SEM) image of Ce-TA polymers showed a spherical morphology with a diameter of ~200 nm (Figure 2b). After calcination at 400 °C, the mesoporous structure and hollow structure were observed in the CeO_2_ nanozyme (Figure 2c and Figure S1). These hollow spheres were produced due to the severe shrinkage of Ce-TA polymers during the calcination process. After the deposition of Au nanoparticles, the morphology of the Au–CeO_2_ nanozyme did not change significantly (Figure 2d,e). Element mapping analysis demonstrated a remarkably homogeneous distribution of Au, O, and Ce, confirming the effective synthesis of the Au–CeO_2_ nanozyme (Figure 2f). The high-resolution transmission electron microscopy images further displayed lattice fringes of 0.270 and 0.319 nm corresponding to the crystal planes of CeO_2_ (100) and (111), as well as 0.235 nm corresponding to the planes of Au (111) (Figure 2g).

**Figure 3:**
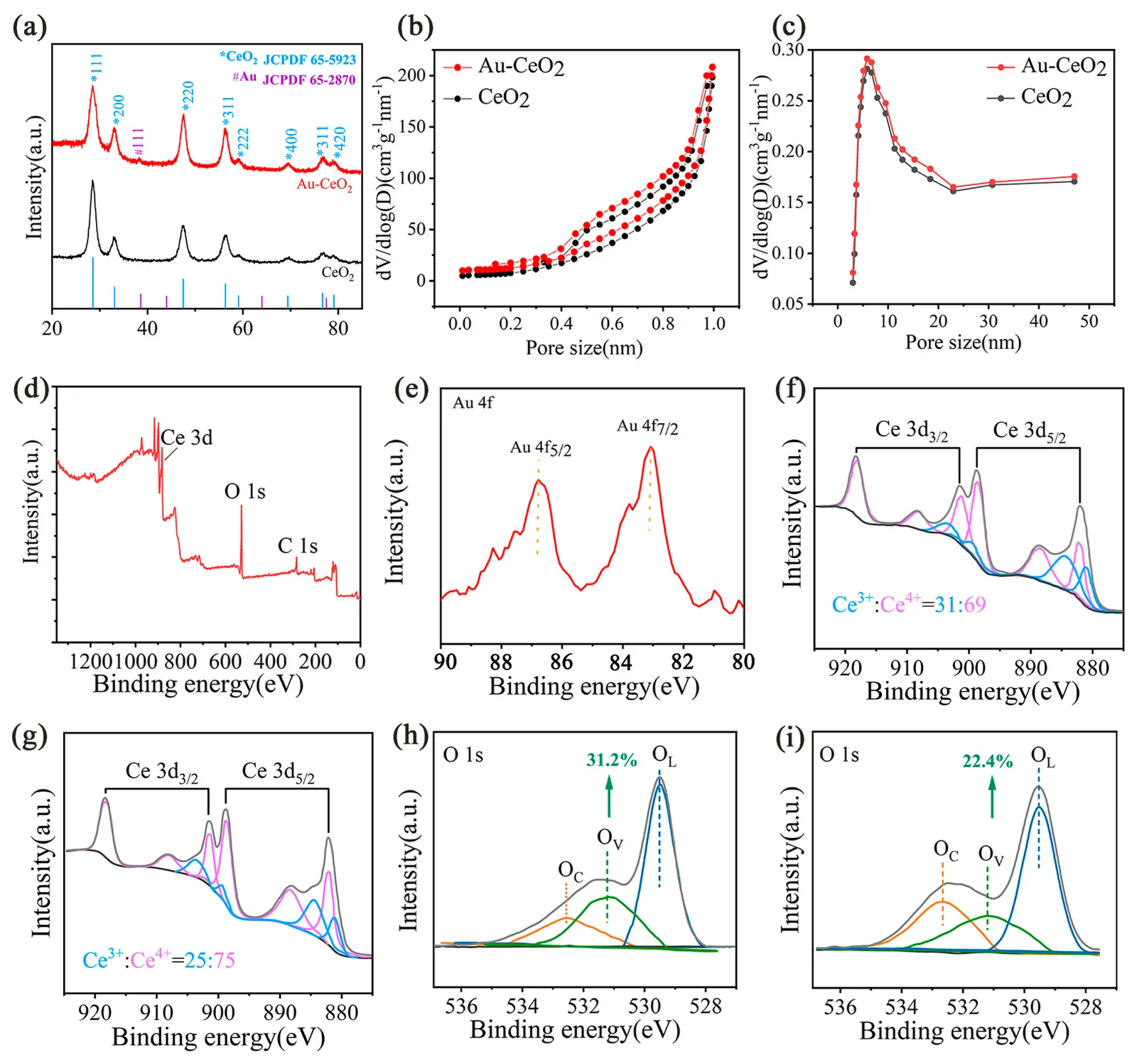
(**a**) XRD pattern of CeO_2_ and Au–CeO_2_ nanozyme. (**b**) N_2_ sorption isotherms and (**c**) pore size distributions for CeO_2_ and Au–CeO_2_ nanozyme (**d**) XPS survey spectrum of Au–CeO_2_ nanozyme. (**e**) Au 4f XPS spectra of Au–CeO_2_ nanozyme. (**f**,**g**) Ce 3d XPS spectra of Au–CeO_2_ and CeO_2_ nanozyme. (**h**,**i**) O 1s XPS spectra of Au–CeO_2_ and CeO_2_ nanozyme.

The crystalline and phase information of the Au–CeO_2_ nanozyme was investigated using X-ray diffraction (XRD) patterns. The patterns revealed the characteristic (111), (200), (220), (311), and (400) planes, which were attributed to the face-centered cubic structure of CeO_2_ (Figure 3a). Additionally, the representative diffraction peaks of Au(111) were obtained, demonstrating the formation of a heterostructure. The specific surface area of the mesoporous Au–CeO_2_ and CeO_2_ nanozyme were 30.5 and 33.8 m^2^/g, respectively (Figure 3b). The pore size of both mesoporous Au–CeO_2_ and CeO_2_ nanozymes was approximately 5.8 nm (Figure 3c). The pore size did not show significant changes, indicating that the appropriate modification with Au did not disrupt the mesoporous structure of the material.

XPS was utilized to analyze the elemental and chemical state distribution within the Au–CeO_2_ nanozyme. The Au 4f spectra exhibited two peaks at 86.7 and 83.1 eV, which were respectively attributed to Au 4f_5/2_ and Au 4f_7/2_ in the 0-valent singlet state (Figure 3d,e) [28]. This further confirms the presence of Au in the Au–CeO_2_ nanozyme. The spectra of Ce 3d established the mixed valence of Ce^3+^ and Ce^4+^ in the Au–CeO_2_ and CeO_2_ nanozymes. The Ce^3+^/Ce^4+^ ratio of the CeO_2_ nanozyme was 25:75, while the Ce^3+^/Ce^4+^ ratio of the Au–CeO_2_ nanozyme was 31:69 (Figure 3f,g). The introduction of Au increased the proportion of Ce^3+^, which could enhance the electron transfer between Ce^3+^ and Ce^4+^ in the Au–CeO_2_ nanozyme. The O1s XPS spectrum was decomposed into three Gaussian components centered at 529.5, 531.2, and 532.8 eV, which were assigned to lattice oxygen (O_L_), oxygen vacancies (O_V_), and chemisorbed and dissociated oxygen species (O_C_), respectively [29]. Following Au loading, the relative proportion of O_V_ rose from 22.4% to 31.2%, thereby enhancing reactant adsorption capacity and boosting overall catalytic performance (Figure 3h,i). Au nanoparticles loaded on the surface of CeO_2_ gave rise to an interfacial electronic effect, which is associated with the increased Ce^3+^ proportion and oxygen vacancy density, consistent with previously reported Au–CeO_2_ systems [28,29,30]. This charge redistribution decreases the energy necessary for the desorption of oxygen atoms, thus leading to a higher concentration of O_V_ at the interface [28,29,30].

### 3.2. Peroxidase-like Activity of Au–CeO_2_ Nanozyme

**Figure 4:**
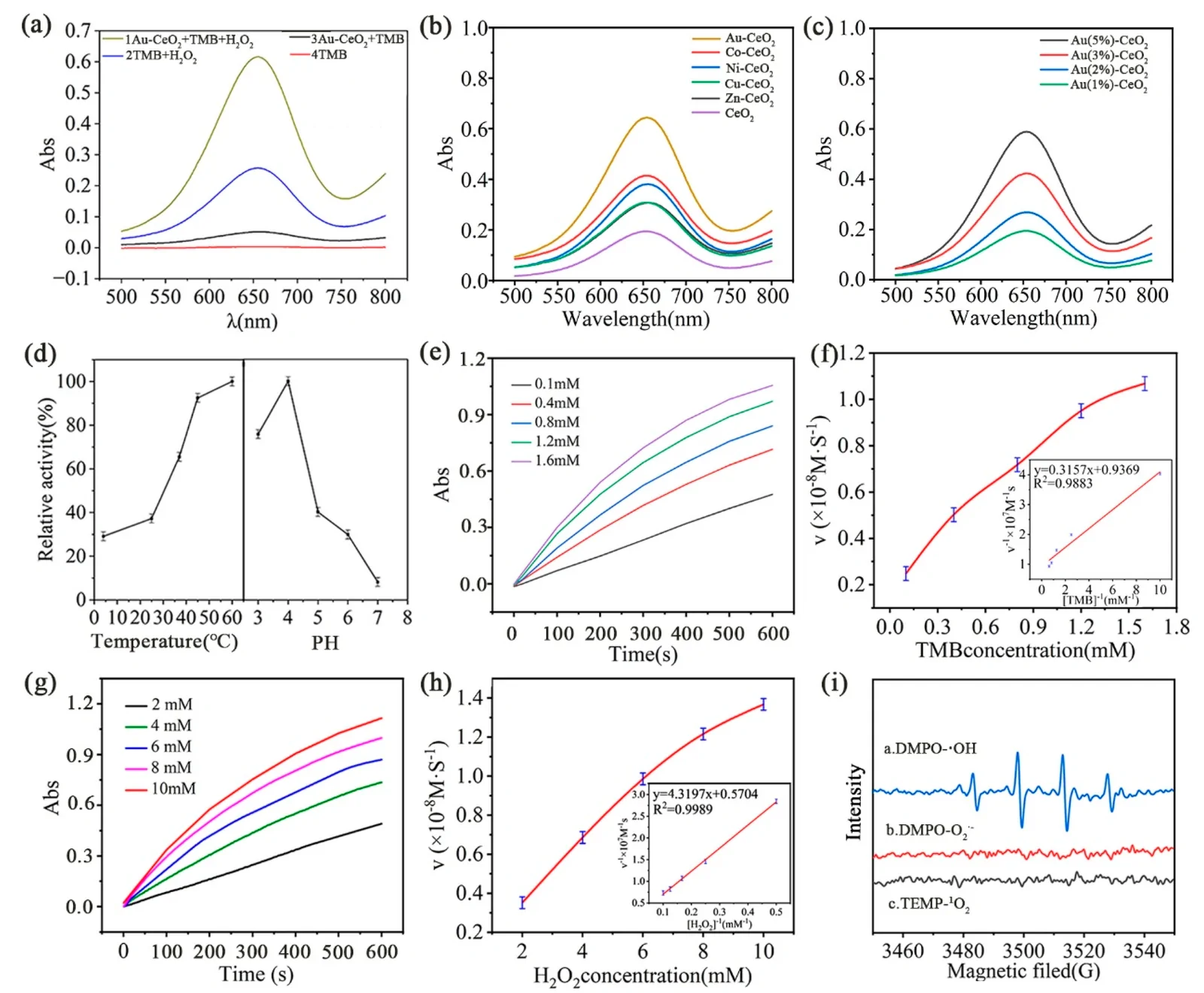
(**a**) UV–vis spectra recorded for various reaction systems The concentrations of CeO_2_ and Au–CeO_2_ nanozyme were both 0.5 mg/mL, TMB and H_2_O_2_ were used at 0.5 mM and 5 mM, respectively. (**b**) UV–vis spectra of TMB/H_2_O_2_ system in the presence of different nanozymes. The concentrations of all nanozyme were 0.5 mg/mL, TMB and H_2_O_2_ were used at 0.5 mM and 5 mM, respectively. (**c**) UV–vis absorption spectra of TMB/H_2_O_2_ with Au–CeO_2_ nanozymes synthesized using various mass ratios of Au. (**d**) The effect of temperature (0–60 °C) and pH (3–7) on the relative catalytic activity of Au–CeO_2_. (**e**) Michaelis–Menten curves and (**f**) the Lineweaver–Burk plots fit for varied concentrations of TMB (0.1–0.5 mM) with fixed concentration of H_2_O_2_ (5 mM). (**g**) Michaelis–Menten curves and (**h**) the Lineweaver–Burk plots fit for varied concentrations of H_2_O_2_ (1–5 mM) with fixed concentration of TMB (0.5 mM). (**i**) EPR signals of DMPO-·OH, and TEMP-^1^O_2_, and DMPO-O_2_^•−^adducts in the presence of Au–CeO_2_ nanozyme and 0.1 M H_2_O_2_.

To explore the peroxidase (POD)-like catalytic activity of the Au–CeO_2_ nanozyme, TMB was employed as a chromogenic substrate. The color of the TMB solution changes from colorless to blue (oxTMB) only in the presence of Au–CeO_2_ nanozyme and H_2_O_2_, as indicated by the characteristic absorbance peak at 652 nm (Figure 4a). The Au–CeO_2_ nanozyme exhibited significant improvement in POD-like activity compared to the CeO_2_ nanozyme (Figure 4a). This enhancement was likely due to the relatively high Ce^3+^/Ce^4+^ ratio in the Au–CeO_2_ nanozyme, which led to stronger and the generation of more ·OH. Meanwhile, the mesoporous structure of Au–CeO_2_ nanozyme effectively enhanced the interaction between catalytic substrates and the active sites. This enhancement is partly attributed to the mesoporous architecture, which may facilitate substrate enrichment and reduce mass-transfer limitations through nanoconfinement effects, as reported in previous studies [34]. Meanwhile, the POD-like activity of CeO_2_ loaded with different metal and Au–CeO_2_ nanozyme were compared. The Au–CeO_2_ nanozyme exhibited a significant enhancement in POD-like activity compared to the CeO_2_ nanozyme loaded with other metals (Figure 4b). When Au nanoparticles were deposited on CeO_2_, a strong metal-support interaction (SMSI) occurred, facilitating the transfer of electrons from Au to Ce^4+^. In contrast, while Co, Cu, Mn, and Ni could also form interfaces with CeO_2_ (e.g., Cu-O-Ce, Co-O-Ce), their capacity to modulate electronic properties was generally less efficient than that of Au. The POD-like activity of the Au–CeO_2_ nanozyme with different mass ratios of Au and CeO_2_ was tested. The Au (5%)–CeO_2_ nanozyme demonstrated the highest peroxidase-like catalytic activity, and subsequent experiments were carried out using this ratio (Figure 4c).

The POD-like activity of Au–CeO_2_ nanozyme was modulated by multiple experimental parameters, including solution pH, reaction temperature, and substrate concentration. Au–CeO_2_ nanozyme demonstrated the optimal catalytic performance at pH 4.0 (Figure 4d). The catalytic performance of Au–CeO_2_ nanozyme increases as the temperature rises (0–60 °C), exhibiting its robust thermal stability (Figure 4d). The steady-state kinetics of Au–CeO_2_ nanozyme were evaluated through the Michaelis–Menten kinetic equation. Au–CeO_2_ nanozyme exhibited maximum reaction rates (*V*_max_) of 1.75 × 10^−7^ M/s for the H_2_O_2_ substrate and 1.04 × 10^−7^ M/s for the TMB substrate, along with low Michaelis constants (*K*_M_) of 7.57 mM and 0.34 mM respectively (Figure 4e–h). The *K*_M_ of CeO_2_ for H_2_O_2_ and TMB were 10.54 and 2.60 mM (Figure S2, Table S1), indicating that Au doping enhances the substrate affinity. Compared with other natural enzymes and nanozymes (Table S1), Au–CeO_2_ nanozyme revealed comparable *V*_max_ and *K*_M_, indicating a rapid catalytic rate and good affinity for both TMB and H_2_O_2_.

To evaluate the stability of Au–CeO_2_ nanozyme in practical applications, we stored the Au–CeO_2_ nanozyme dispersion solution (0.5 mg/mL) at 4 °C and measured its catalytic activity at 0–7 days (Figure S3). The results showed that the relative activity remained above 99.1% of the initial value within the 7-day storage period (RSD < 1.2%, *n* = 3), indicating that the Au–CeO_2_ nanozyme showed good storage stability. Such excellent storage stability is advantageous for practical applications, as it ensures reliable and reproducible detection performance over extended periods.

### 3.3. Peroxidase-like Mechanism of Au–CeO_2_ Nanozyme

To identify the reactive oxygen species (ROS) involved in the catalytic process, electron paramagnetic resonance (EPR) spectroscopy was performed using DMPO and TEMP as spin-trapping agents. As shown in Figure 4i, upon the addition of Au–CeO_2_ nanozyme and H_2_O_2_, the EPR spectrum displayed four characteristic peaks with a 1:2:2:1 intensity ratio, corresponding to the DMPO-·OH adduct. In contrast, no characteristic signals corresponding to DMPO-O_2_·^−^ or TEMP-^1^O_2_ adducts were detected, indicating that ·OH is the predominant ROS responsible for the oxidation of TMB in the Au–CeO_2_/H_2_O_2_ system.

To further elucidate the origin of the enhanced ·OH generation, we compared the EPR signals of pristine CeO_2_ and Au–CeO_2_ under identical conditions (Figure S4). The Au–CeO_2_ nanozyme exhibited a substantially stronger DMPO-·OH signal than pristine CeO_2_, with the integrated signal intensity. This enhancement is consistent with the XPS results (Figure 3f–i), which showed that Au loading increased the Ce^3+^/Ce^4+^ ratio (from 25:75 to 31:69) and oxygen vacancy density (from 22.4% to 31.2%).

**Figure 5:**
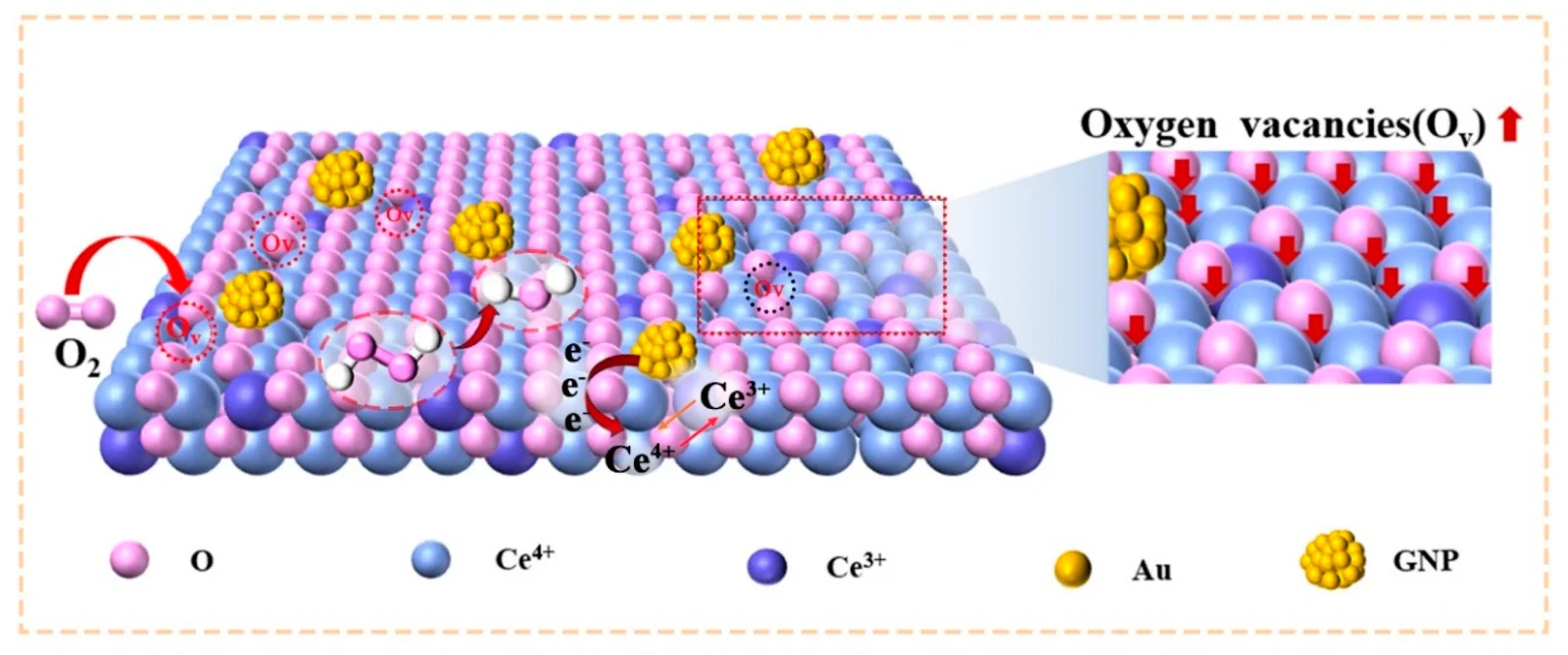
Illustrative scheme outlining the POD-like catalytic activity of the Au–CeO_2_ nanozyme.

Based on these experimental observations and previous reports [3,21,37,38], a plausible catalytic mechanism is proposed (Figure 5). The increased Ce^3+^ content and oxygen vacancies in Au–CeO_2_ are known to facilitate the Fenton-like decomposition of H_2_O_2_ via the following pathway: Ce^3+^ + H_2_O_2_ → Ce^4+^ + ·OH + OH^−^. The elevated concentration of redox-active Ce^3+^ sites provides more electron donors for H_2_O_2_ activation, promoting the sustained generation of highly reactive ·OH species. Meanwhile, oxygen vacancies served as active sites for H_2_O_2_ adsorption and activation, lowering the energy barrier for O–O bond cleavage [39,40,41]. The synergistic effect between Ce^3+^ sites and oxygen vacancies thus contributes to the enhanced peroxidase-like catalytic activity of the Au–CeO_2_ nanozyme. Additionally, the mesoporous architecture is expected to facilitate substrate accessibility to the active sites, which may further support the overall catalytic efficiency, as suggested by previous studies on mesoporous nanozymes [42,43,44].

### 3.4. Ratiometric Colorimetric Detection of NO_2_^−^

**Figure 6:**
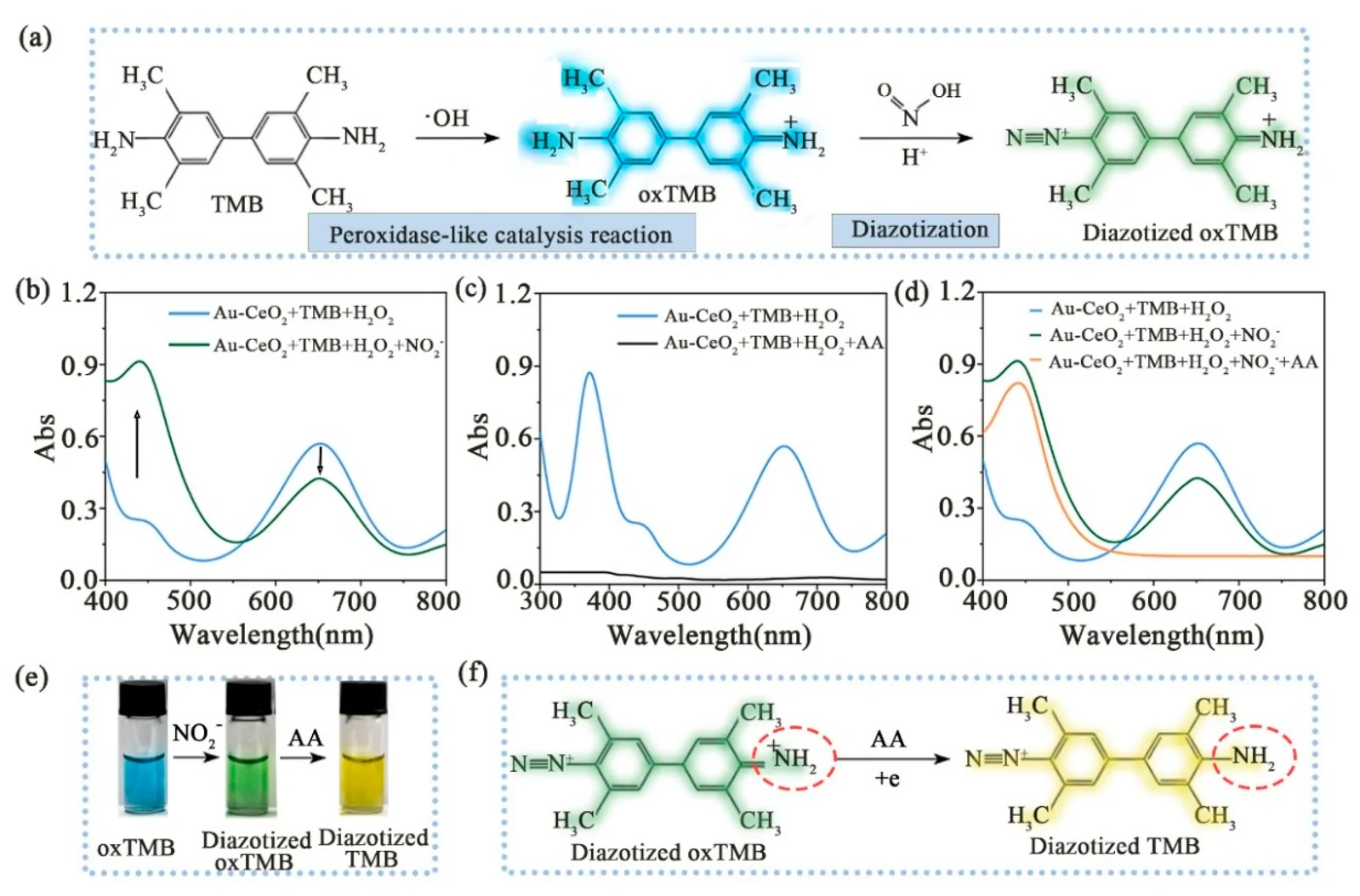
(**a**) Schematic diagram of the oxidation of TMB and the diazotization reaction of oxTMB and NO_2_^−^. (**b**) UV–visible spectroscopy of the Au–CeO_2/_TMB/H_2_O_2_ system with and without NO_2_^−^. (**c**) UV–visible spectroscopy of the Au–CeO_2_/TMB/H_2_O_2_ system with and without AA. (**d**) UV–visible spectroscopy of the Au–CeO_2_/TMB/H_2_O_2_, Au–CeO_2_/TMB/H_2_O_2_/NO_2_^−^ and Au–CeO_2_/TMB/H_2_O_2_/NO_2_^−^/AA system. (**e**) Photographs and (**f**) mechanism of the redox reaction of diazotized oxTMB and AA.

The detection mechanism involves two distinct steps that should be clearly distinguished (Figure 6a). In the first step (signal generation), the Au–CeO_2_ nanozyme catalyzes the H_2_O_2_-mediated oxidation of TMB to oxTMB, producing the blue color with a characteristic absorption at 652 nm. This step is responsible for generating the colorimetric signal but does not provide selectivity toward nitrite. In the second step (selective recognition), NO_2_^−^ reacts specifically with the aromatic primary amine moiety of oxTMB under acidic conditions via diazotization, forming a diazo product with a new absorption band at 445 nm and a concurrent decrease at 652 nm (Figure 6b) [45,46,47].

To verify this conclusion, reduction experiments were conducted using ascorbic acid (AA), which reduced oxTMB back to colorless TMB (Figure 6c). However, in the presence of NO_2_^−^, oxTMB underwent diazotization instead, forming a yellow-colored diazo-coupled product (Figure 6d,e). When AA was subsequently introduced under the condition, only partial reduction occurred. Specifically, the semiquinone imine unit within the diazotized oxTMB was reduced to an aromatic amine (Figure 6d–f). Crucially, the superior thermodynamic stability of the diazo group (–N^+^≡N) and its resistance to reductive cleavage prevented further transformation (Figure 6f) [48,49,50]. This selective preservation of the diazo functionality strongly confirms that diazotization between NO_2_^−^ and oxTMB has indeed occurred. Therefore, these findings demonstrate the ratiometric colorimetric assay based on the Au–CeO_2_/TMB/H_2_O_2_ system is practically feasible for highly sensitive and selective detection of NO_2_^−^.

**Figure 7:**
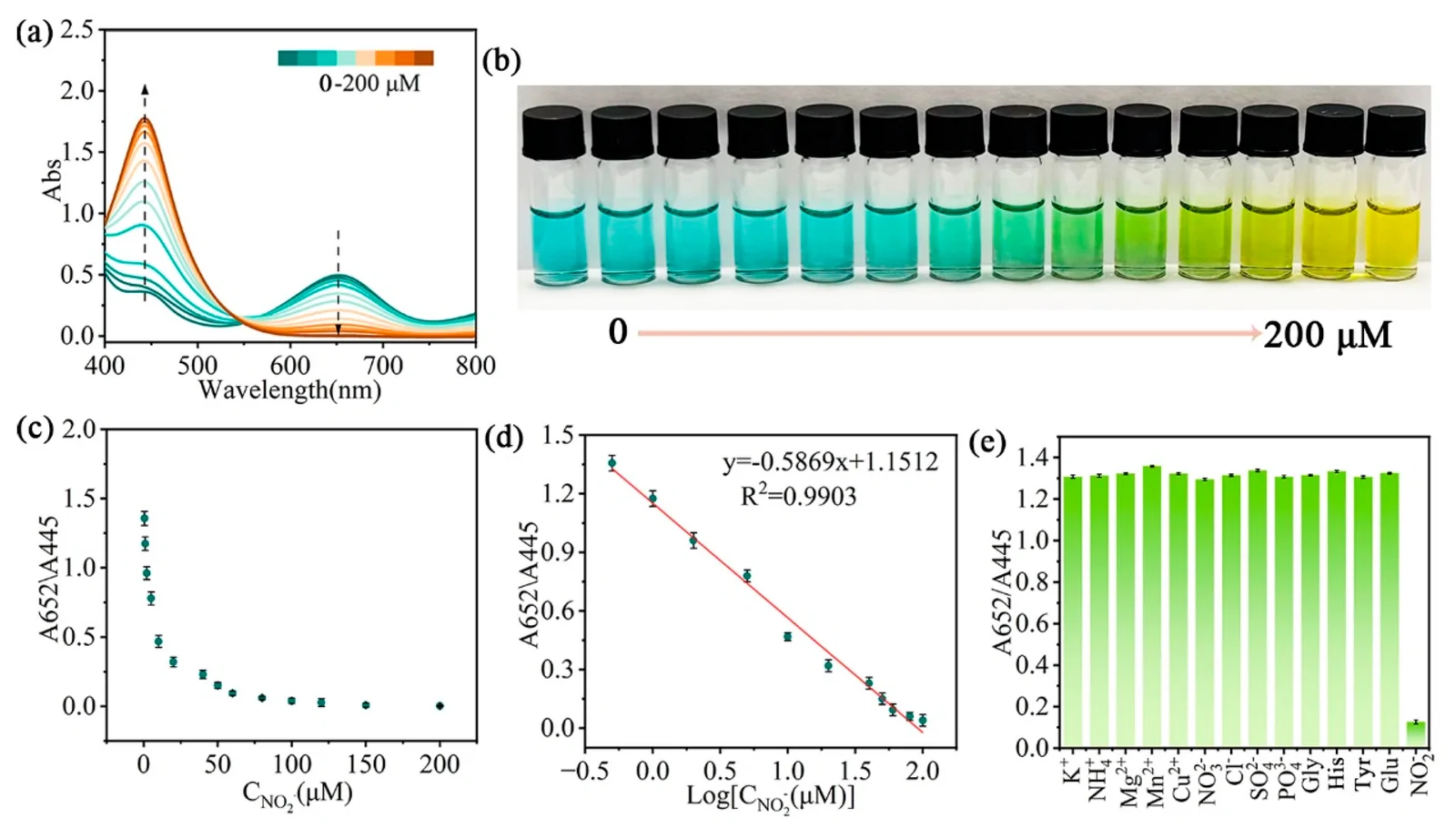
(**a**) UV–vis spectra and photographs (**b**) of the Au–CeO_2_/TMB/H_2_O_2_ system with various concentrations of NO_2_^−^ (1–200 μM). (**c**) Absorbance ratio (A_652_/A_445_) versus the different NO_2_^−^ concentration (1–200 μM). (**d**) The calibration curve correlating the absorbance ratio (A_652_/A_445_) to the NO_2_^−^ concentration. (**e**) The anti-interference performance of the Au–CeO_2_/TMB/H_2_O_2_ system with various different interferences.

The sensitivity of the Au–CeO_2_/TMB/H_2_O_2_ system as a ratiometric colorimetric sensor was assessed by optimizing the diazotization time for oxTMB. The signal ratio (A_652_/A_445_) declined with the increase in reaction time and gradually reached a stable state after 15 min. (Figure S5). As the NO_2_^−^ concentration increased, the color of the system solution transitioned from blue to green, and subsequently to yellow. Accordingly, the absorbance signal at 652 nm gradually decreased, while the signal at 445 nm continuously increased (Figure 7a,b). Consequently, the A_652_/A_445_ ratio declined progressively as the NO_2_^−^ concentration rose (Figure 7c). A linear relationship existed between the ratio A_652_/A_445_ and the logarithm of NO_2_^−^ concentration in the range of 1–200 μM (A_652_/A_445_ = −0.5869 log*C*_Nitrite_ + 1.1512), with the limit of detection (LOD = 3σ/S) of 0.43 μM (Figure 7d). This ratiometric colorimetric sensor exhibited detection limits and ranges that are comparable to those of previously reported methods (Table S2), which suggested its significant potential for practical applications. To elucidate the contribution of Au loading to the sensing performance, we compared the analytical performance of pristine CeO_2_ and Au–CeO_2_ nanozymes for nitrite detection under identical conditions (Figure S6a). A linear relationship existed between the ratio A_652_/A_445_ and the logarithm of NO_2_^−^ concentration in the range of 30–200 μM (A_652_/A_445_ = −3.4819 log*C*_Nitrite_ + 7.9401), with the limit of detection (LOD = 3σ/S) of 12.5 μM (Figure S6b,c). This substantial improvement can be attributed to the increased Ce^3+^/Ce^4+^ ratio and oxygen vacancy density in Au–CeO_2_ (Figure 3f–i), which facilitate ·OH generation and promote the diazotization reaction between nitrite and oxTMB. These results unequivocally demonstrate that Au loading plays a crucial role in enhancing both the catalytic activity and the sensing performance of the CeO_2_ nanozyme.

To evaluate whether the ratiometric detection mode offers an analytical advantage over single-wavelength measurements, the three detection modes (A_652_, A_445_, and A_652_/A_445_) were compared under simulated matrix background conditions (Figure S7). Crystal violet was added at three concentration levels to mimic the background absorbance of colored food matrices, and the NO_2_^−^ concentration was back-calculated using each detection mode after background subtraction (Table S3). At low background, all three modes gave recoveries close to 100%. As the background level increased, the recoveries obtained from the single-wavelength detection mode deviated progressively from 100% (93.6–104.3% for A_445_ and 93.8–105.9% for A_652_), whereas the ratiometric detection mode recoveries of 99.6–102.3% with lower RSDs (1.06–1.72%). These results indicate that, although A_652_ and A_445_ are not independent channels, the ratio partially cancels proportional background effects and thus provides a measurable advantage in accuracy and precision under colored-background conditions.

In addition to sensitivity, we also assessed the selectivity of this ratiometric colorimetric method. The influences of common substances such as metal ions, inorganic ions, and organic small molecules on NO_2_^−^ determination were negligible, indicating that the ratiometric colorimetric assay exhibits excellent anti-interference ability for NO_2_^−^ detection (Figure 7e). This high selectivity is mainly attributed to the strong catalytic effect of the Au–CeO_2_ nanozyme on the TMB chromogenic reaction and the specific diazotization interaction between oxTMB and NO_2_^−^.

### 3.5. Assay of NO_2_^−^ for Real Samples

To evaluate the practicality of the ratiometric colorimetric sensor, quantitative analysis was conducted on NO_2_^−^ concentrations based on the standard addition assay in fermented vegetables and salted duck eggs. After adding 5–30 μM of NO_2_^−^ to these food samples, the recovery rates ranged from 95.4% to 106.6%, with a relative standard deviation (RSD) between 1.02% and 2.73%. This indicates that the method is highly reliable for food sample analysis. To further improve the accuracy of this colorimetric method for detecting NO_2_^−^, we used a commercially available test kit based on the Griess reaction as a reference tool (GB 5009.33-2016) to compare. The linear detection equation for the commercial kit was Y = 0.01265 + 0.00063X (R^2^ = 0.974), with a detection range of 3–32 μM (Figure S8). The detection results from the standard method were highly consistent with those from our proposed method (Table S4). It should be noted that the Griess method was used here as a reference method to validate the accuracy of the proposed assay, rather than as a benchmark for performance comparison; the two methods are based on different signal generation mechanisms and are suited to different analytical scenarios. To challenge the method under strongly colored conditions, soy sauce extract was selected as an additional matrix. After dilution and matrix-blank correction, the recoveries at three spiked levels (5, 10, and 30 μM) were 96.4–103.8%, with RSDs of 1.67–2.86% (Table S4). These values were comparable to those obtained in the less intensely colored matrices (fermented vegetables and salted duck eggs), indicating that the combination of dilution, matrix-blank subtraction, and matrix-matched calibration effectively mitigates background interference at the concentration levels tested. These results collectively demonstrate the practicality of our method and highlight its broad application potential in fields such as food safety management.

## 4. Conclusions

In this work, we successfully developed a mesoporous Au–CeO_2_ nanozyme with significantly enhanced peroxidase-like activity through a simple and scalable synthetic strategy. The introduction of Au nanoparticles increased the Ce^3+^/Ce^4+^ ratio (from 25:75 to 31:69) and oxygen vacancy density (from 22.4% to 31.2%), collectively facilitating reactive ·OH generation and improving catalytic efficiency. Meanwhile, the mesoporous structure effectively enhanced the interaction between catalytic substrates and the active sites of nanozymes. The mesoporous structure likely promotes substrate accessibility to the active sites, contributing to the enhanced catalytic performance. By leveraging this nanozyme, we established a ratiometric colorimetric assay for nitrite detection based on the Au–CeO_2_-catalyzed oxidation of TMB and the subsequent diazotization reaction between nitrite and oxTMB. The system exhibited a low detection limit of 0.43 μM and outstanding selectivity against common interferents. Moreover, the method was successfully applied to detect nitrite in fermented vegetables and salted duck eggs with satisfactory recoveries (95.4–106.6%, RSD < 2.73%). The test results demonstrated good correlation with the standard Griess-based kit. These results highlight the practical utility of the Au–CeO_2_ nanozyme-based ratiometric colorimetric assay for rapid, cost-effective, and reliable nitrite monitoring in complex food matrices.

## Data Availability

The original contributions presented in this study are included in the article/supplementary material. Further inquiries can be directed to the corresponding author.

## Supplementary Materials

The following supporting information can be downloaded at: https://www.mdpi.com/article/10.3390/foods15213881/s1. Figure S1: (a) TEM images of CeO_2_. (b) Element mapping of CeO_2_. Figure S2: (a) Absorbance versus reaction time with different concentrations of H_2_O_2_: 0.5 mg/mL CeO_2_ nanozyme, 1-5 mM H_2_O_2_, 0.5 mM TMB. (b) The corresponding steady-state kinetic assays of CeO_2_ nanozyme for H_2_O_2_. (c) Linear double-reciprocal plotting for CeO_2_ nanozyme with H_2_O_2_ as substrate. (d) Absorbance versus reaction time with different concentrations of TMB: 0.5 mg/mL CeO_2_ nanozyme, 0.1-0.5 mM TMB, 5 mM H_2_O_2_. (e) The corresponding steady-state kinetic assays of CeO_2_ nanozyme for TMB. (f) Linear double-reciprocal plotting for CeO_2_ nanozyme with TMB as substrate. Figure S3: The storage stability of Au-CeO_2_ nanozyme at 4 °C over 7 days. Figure S4: EPR signals of DMPO-·OH adducts in the presence of Au-CeO_2_ nanozyme + H_2_O_2_. and CeO_2_ nanozyme + H_2_O_2_. The concentrations of the Au-CeO_2_ nanozyme and CeO_2_ nanozyme were 1 mg/mL, and the concentration of H_2_O_2_ was 0.1 M. Figure S5: The ratiometric colorimetric signal of the Au-CeO_2_/TMB/H_2_O_2_ system with NO_2_^−^ (100 μM) over reaction time. Figure S6: (a) UV–vis spectra of the CeO_2_/TMB/H_2_O_2_ system with various concentrations of NO_2_^−^ (10-300 μM). (b) Absorbance ratio (A_652_/A_445_) versus the different NO_2_^−^ concentration (10-300 μM). (c) The calibration curve correlating the absorbance ratio (A_652_/A_445_) to the NO_2_^−^ concentration (30-200 μM). Figure S7: (a-b) The calibration curve correlating the absorbance A_652_ and A_445_ to the NO_2_^−^ concentration. Figure S8: The linear relationship between commercial kits and NO_2_^—^ concentrations. Table S1: Comparison of catalytic kinetic constants. Table S2: Performance comparison of various nanozymes for nitrite detection. Table S3: Comparison of recovery and precision obtained with (A_652_, A_445_ and A_652_/A_445_) under simulated background interference at different levels (n = 3). Table S4: Detection results of NO^2−^ content in actual samples by ratiometric colorimetric method (n = 3). [3,4,18,28,51,52,53,54,55,56,57,58,59,60,61,62,63,64,65].
