# Supplementary material for: The Au–CeO2 Nanozyme with Enhanced Peroxidase-like Activity for Highly Sensitive Ratiometric Colorimetric Detection of Nitrite
Source: Foods. 2026 Oct 29;15(21):3881. doi: 10.3390/foods15213881 (PMC13762645; doi:10.3390/foods15213881)
Supplement: Supplementary file 1 [file foods-15-03881-s001.zip › foods-4599060-supplementary.pdf]

Supporting Information

The Au-CeO_2 Nanozyme with Enhanced Peroxidase-Like Activity for Highly Sensitive Ratiometric Colorimetric Detection of Nitrite

Luyu Chen [1], Wenting Lu [1], Chunyan Sun [1], Yingrui Zhang [1], Jicai Cai [1], Jiaqi Chen [1], Linna Du [1], Jiajun Hu [2], and Jing Qin[1, 3]*

1 College of Advanced Materials Engineering, Jiaxing Nanhu university, Jiaxing, 314001, China; 19817626673@163.com (L.Y.C.); 18168842670@163.com (W.T.L.); 13663780247@163.com (C.Y.S.); 17357350817@163.com (Y.R.Z); 15236911235@163.com (J.C.C.); cherry_0412@foxmail.com (J.Q.C.); linna-du1983@jxnhu.edu.cn (L.N.D); jingqin@jxnhu.edu.cn (J.Q.)

2 Zhejiang Huayou Cobalt Co., Ltd, Jiaxing, 314599, China; hujiajun@huayou.com (J.J.H)

3 Jiaxing Nanhu university − Zhejiang Wufangzhai Industrial Co., Ltd. Green Food Technology Innovation La-boratory, Jiaxing, 314001, China. jingqin@jxnhu.edu.cn (J.Q.)

* Correspondence: jingqin@jxnhu.edu.cn (J.Q.)

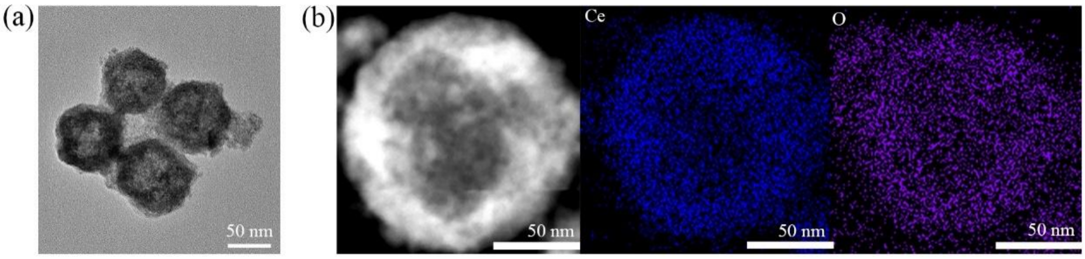

Figure S1 (a) TEM images of CeO_2. (b) Element mapping of CeO_2.

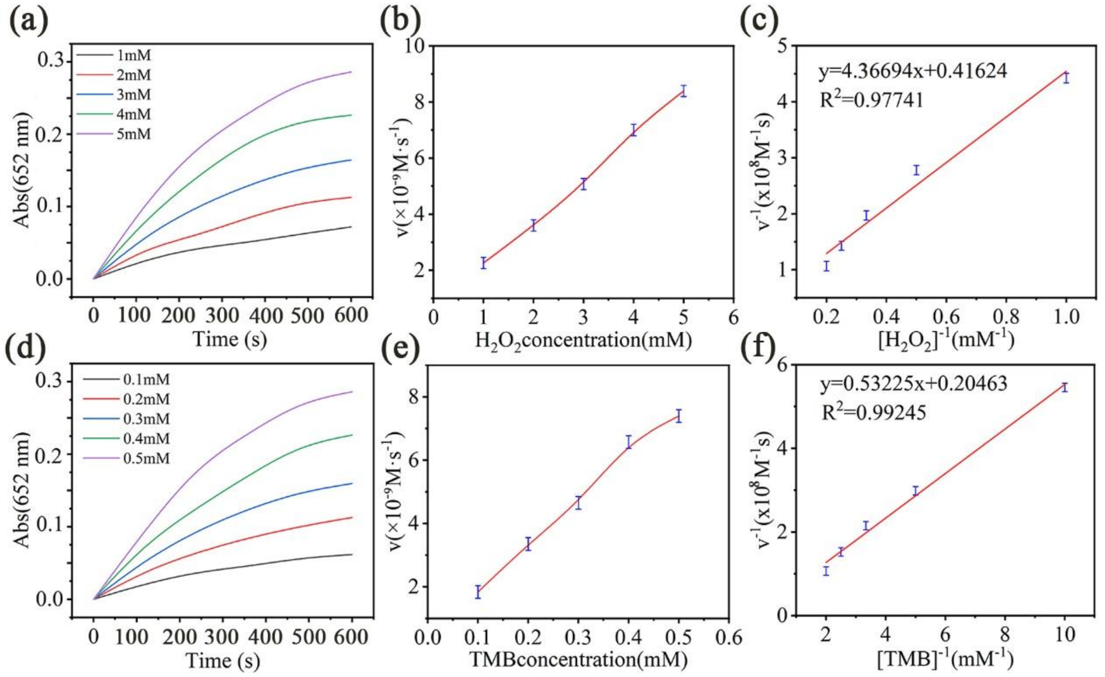

Figure S2. (a) Absorbance versus reaction time with different concentrations of H_2O_2: 0.5 mg/mL CeO_2 nanozyme, 1-5 mM H_2O_2, 0.5 mM TMB. (b) The corresponding steady-state kinetic assays of CeO_2 nanozyme for H_2O_2. (c) Linear double-reciprocal plotting for CeO_2 nanozyme with H_2O_2 as substrate. (d) Absorbance versus reaction time with different concentrations of TMB: 0.5 mg/ mL CeO_2 nanozyme, 0.1-0.5 mM TMB, 5 mM H_2O_2. (e) The corresponding steady-state kinetic assays of CeO_2 nanozyme for TMB. (f) Linear double-reciprocal plotting for CeO_2 nanozyme with TMB as substrate.

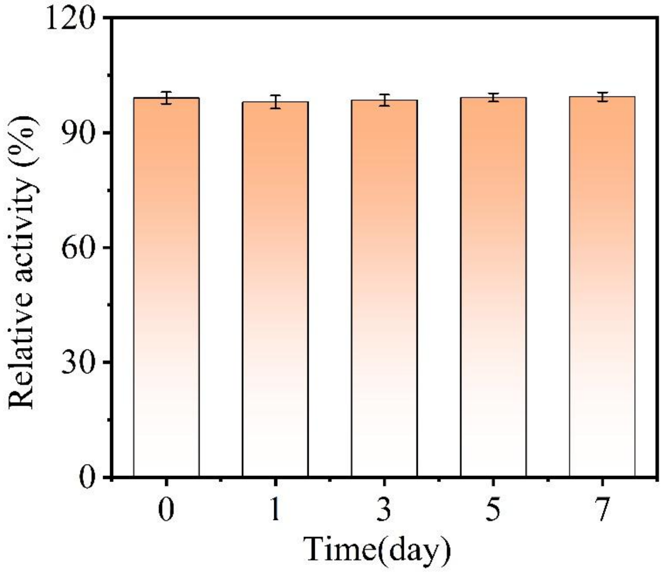

Figure S3 The storage stability of Au-CeO_2 nanozyme at 4°C over 7 days.

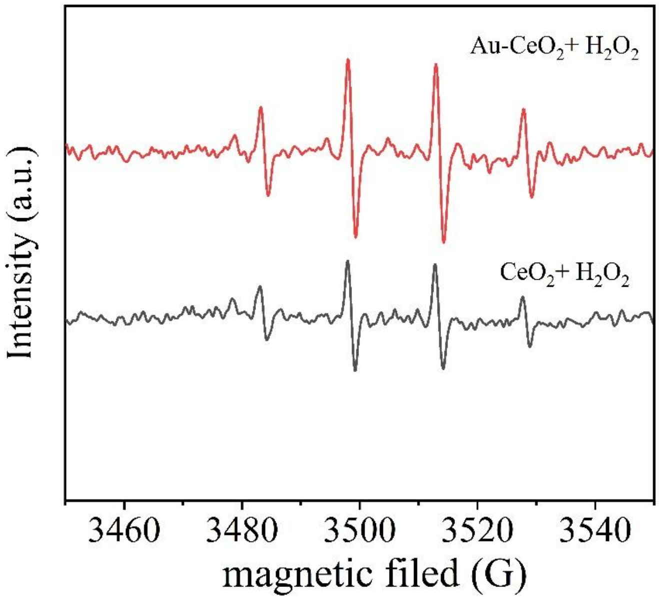

Figure S4 EPR signals of DMPO-·OH adducts in the presence of Au-CeO$_2$ nanozyme + H_2O_2. and CeO$_2$ nanozyme + H_2O_2. The concentrations of the Au-CeO$_2$ nanozyme and CeO$_2$ nanozyme were 1 mg/mL, and the concentration of H_2O_2 was 0.1 M

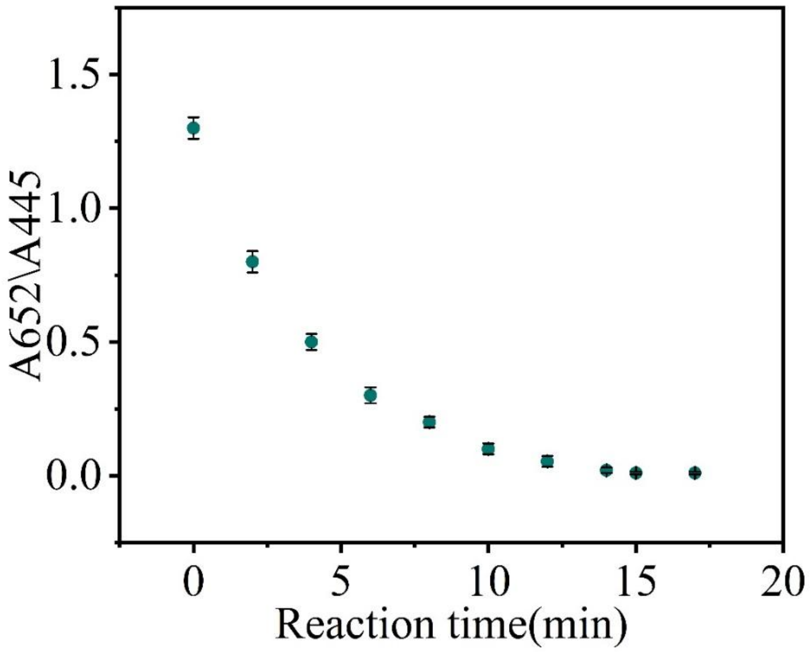

Figure S5 The ratiometric colorimetric signal of the Au-CeO_2/TMB/H_2O_2 system with NO_2^- (100 μM) over reaction time.

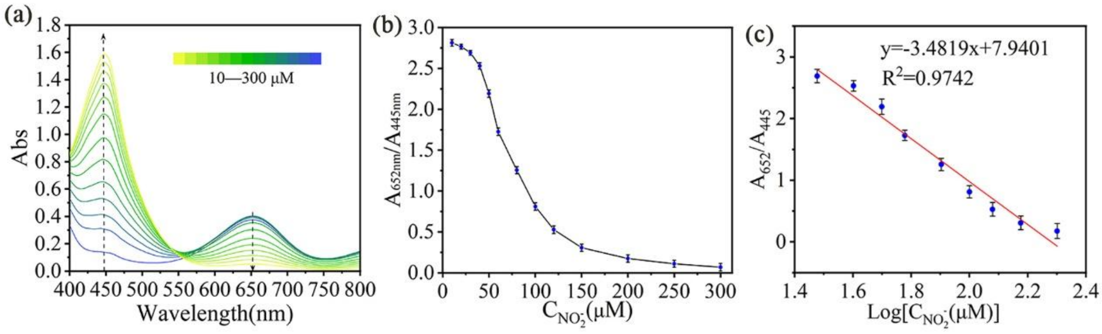

Figure S6 (a) UV–vis spectra of the CeO_2/TMB/H_2O_2 system with various concentrations of NO_2^- (10-300 μM). (b) Absorbance ratio (A_{652}/A_{445}) versus the different NO_2^- concentration (10-300 μM). (c) The calibration curve correlating the absorbance ratio (A_{652}/A_{445}) to the NO_2^- concentration (30-200 μM).

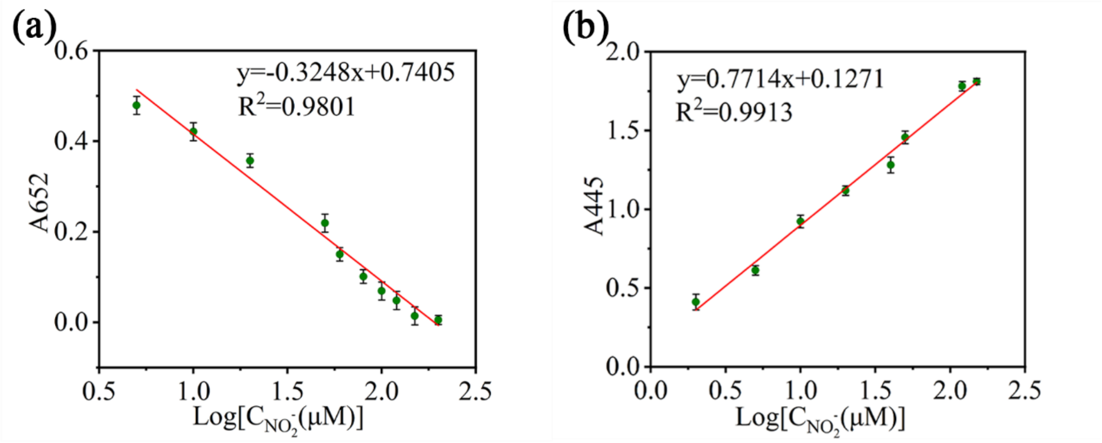

Figure S7 (a-b) The calibration curve correlating the absorbance A_{652} and A_{445} to the NO_2^- concentration.

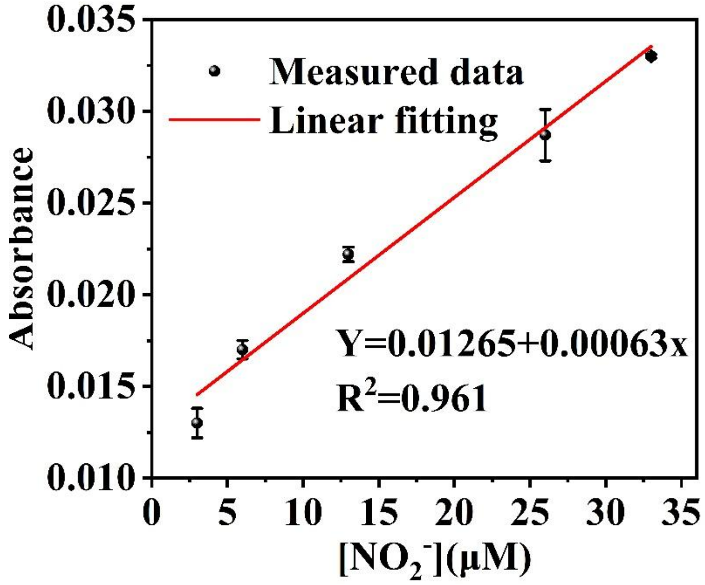

Figure S8 The linear relationship between commercial kits and NO_2^- concentrations

Table S1 Comparison of catalytic kinetic constants

Sample	K_M (mM)		V_{max} (M s^{-1})		Ref.
	H_2O_2	TMB	H_2O_2	TMB	
HRP	3.7	0.43	8.7×10^{-8}	1×10^{-7}	[1]
CeO_2	28.24	0.285	5.07×10^{-7}	7.9×10^{-9}	[2]
Pt/CeO_2	16.18	0.408	1.08×10^{-10}	8.84×10^{-9}	[3]
Au/CeO_2	20	0.15	8.60×10^{-8}	4.45×10^{-8}	[4]
$Pd_{1.7}Bi@CeO_2$	2.99	_	2.16×10^{-8}	_	[5]
Ceria	176	0.78	4×10^{-5}	1.9×10^{-5}	[6]
Au/CeO_2	22.5	0.055	5.3×10^{-8}	9.5×10^{-8}	[7]
CeO_2	10.54	2.60	2.4×10^{-8}	4.89×10^{-8}	This work
$Au\text{-}CeO_2$	7.57	0.34	1.75×10^{-7}	1.04×10^{-7}	This work

Table S2 Performance comparison of various nanozymes for nitrite detection

Sample	Method	Linear range	LOD (μM)	Ref
AuNP-CeO_2NP@GO	Colorimetric	100-5000 μM	4.6	[8]
MoO_3	Colorimetric	0-30 μM	0.1	[9]
V-Fe_3O_4	Colorimetric	3-350	2.22	[10]
Nap-FF	Colorimetric	8.3μM -16.7 mM	0.72	[11]
MnFeO	Colorimetric	3.33-133.3 μM	0.2	[12]
NED-grafted cellulose	Colorimetric	0.5-50μM	0.29	[13]
(Fe SACs)	Colorimetric	1–120 μM	0.238	[14]
Mn/Yb/Er-CeO_2	Colorimetric	2–100 μM	0.29	[15]
$PtCu_3$	Colorimetric	5–500 μM	1.94	[16]
N-CDs	Colorimetric	5–100 μM	1.36	[17]
Mn_3O_4/C	Colorimetric	3–150 μM	0.4	[18]
CoOOH	Colorimetric	2–175 μM	2.04	[19]
Au-CeO_2	Colorimetric	1–200 μM	0.43	This work

Table S3 Comparison of recovery and precision obtained with (A_{652}, A_{445} and A_{652}/A_{445}) under simulated background interference at different levels (n = 3)

.

Background level	Spiked NO_2^- (μM)	A_{445}		A_{652}		A_{652}/A_{445}	
		Recovery (%)	RSD (%)	Recovery (%)	RSD (%)	Recovery (%)	RSD (%)
no	5	99.54	1.91	99.31	1.76	99.97	1.07
low	5	98.4	1.62	93.8	1.56	99.6	1.06
medium	5	93.6	2.96	105.9	2.91	102.3	1.72
high	5	104.3	2.64	97.4	2.38	100.6	1.54

* The low, medium and high levels of background represent the concentrations of crystal violet as 0.05, 0.1 and 0.5 mg/mL respectively.

Table S4 Detection results of NO_2^- content in actual samples by ratiometric colorimetric method (n = 3)

Sample	Added (μM)	This work Detected (μM)	Recovery (%)	RSD (%)	Griess-based kit Detected (μM)	Recovery (%)	RSD (%)
fermented vegetables	0	6.12	—	1.08	6.08	—	1.12
	5.00	10.89	95.4	1.02	10.96	97.6	1.16
	10.0	16.78	106.6	2.16	16.47	103.9	0.91
	30.0	36.35	100.7	2.04	36.15	100.2	2.38
salted duck eggs	0	4.87	—	2.01	5.12	—	1.35
	5.00	9.66	95.8	2.73	9.98	97.2	1.45
	10.0	15.15	102.8	1.38	15.09	99.7	2.53
	30.0	34.56	98.9	2.33	35.37	100.8	1.72
soy sauce	0	3.42	—	1.71	3.36	—	1.25
	5.00	8.24	96.4	2.86	8.17	96.2	1.65
	10.0	13.8	103.8	1.67	13.23	98.7	2.43
	30.0	33.69	100.9	1.73	33.9	101.8	1.62

.

References

[1] M. Gao, X. Lu, S. Chen, D. Tian, Y. Zhu, C. Wang. Enhanced Peroxidase-like Activity of Mo^{6+}-Doped Co_3O_4 Nanotubes for Ultrasensitive and Colorimetric l-Cysteine Detection. *ACS Appl. Nano Mater.*, 2018, **1**(9), 4703-4715.

[2] J. Qin, Y. Y. Feng, D. Cheng, B. W. Liu, Z. Wang, Y. X. Zhao, J. Wei. Construction of a Mesoporous Ceria Hollow Sphere/Enzyme Nanoreactor for Enhanced Cascade Catalytic Antibacterial Therapy. *ACS Appl. Mater. Interfaces*, 2021, **13**(34), 40302-40314.

[3] Liu, X.; Wang, X.; Qi, C.; Han, Q.; Xiao, W.; Cai, S.; Wang, C.; Yang, R. Sensitive Colorimetric Detection of Ascorbic Acid Using Pt/CeO_2 Nanocomposites as Peroxidase Mimics. *Appl. Surf. Sci.* 2019, **479**, 532–539.

[4] Y. Li, P. Li, M. Qi, X. Zhang, J. Meng. SERS-Based Non-Enzymatic Detection of Uric Acid Using Au/CeO_2 Nanorods. *ACS Appl. Nano Mater.*, 2024, **7**, 20920-20932.

[5] X. Chen, C. Zhao, D. Liu, K. Lin, J. Lu, S. Zhao, J. Yang, H. Lin. Intelligent Pd1.7Bi@CeO2 Nanosystem with Dual-Enzyme-Mimetic Activities for Cancer Hypoxia Relief and Synergistic Photothermal /Photodynamic/Chemodynamic Therapy. *ACS Appl. Mater. Interfaces*, 2023, **15,** 21804-21818.

[6] Y. Chen, C. Yin, Y. Zhang, Y. Liu, J. Zan, C. Xie, Q. Fan, W. Huang. "Dual lock-and-key"-controlled ceria nanotubes-based nanozymes for tumor-specific photothermal therapy. *Dyes Pigm.*, 2021, **191**, 109350.

[7] B. Yang, J. Y. Zeng, G. X. Zhao, C. P. Ding, L. Chen, Y. J. Huang. Cascade enzyme-mimicking with spatially separated gold-ceria for dual-mode detection of superoxide anions. *Biosens. Bioelectron.*, 2025, **267**, 116847.

[8] O. Adegoke, S. Zolotovskaya, A. Abdolvand, N. N. Daeid. Rapid and highly selective colorimetric detection of nitrite based on the catalytic-enhanced reaction of mimetic Au nanoparticle-CeO_2 nanoparticle-graphene oxide hybrid nanozyme. *Talanta*, 2021, **224**, 121875.

[9] J. Du, M. Zhao, W. Huang, Y. Deng, Y. He. Visual colorimetric detection of tin(II) and nitrite using a molybdenum oxide nanomaterial-based three-input logic gate. *Anal. Bioanal. Chem.*, 2018, **410**(18), 4519-4526.

[10] R. Pol, L. Diez, D. Gabriel, M. Baeza. Versatile Three-Dimensional-Printed Platform for Nitrite Ion Analyses Using a Smartphone with Real-Time Location. *Anal. Chem.*, 2019, **91**(21), 13916-13923.

[11] Y. T. Tai, C. Y. Cheng, Y. S. Chen, F. H. Ko. A hydrogel-based chemosensor applied in conjunction with a Griess assay for real-time colorimetric detection of nitrite in the environment. *Sens. Actuators B Chem.*, 2022, **369**, 132298.

[12] M. Wang, P. Liu, H. Zhu, B. Liu, X. Niu. Ratiometric Colorimetric Detection of Nitrite Realized by Stringing Nanozyme Catalysis and Diazotization Together. *Biosensors*, 2021, **11**(8), 280.
[13] T. L. Mako, A. M. Levenson, M. Levine. Ultrasensitive Detection of Nitrite through Implementation of N-(1-Naphthyl)ethylenediamine-Grafted Cellulose into a Paper-Based Device. *ACS Sens.*, 2020, **5**(4), 1207-1215.
[14] J. Liu, L. Gong, H. Chen, J. Gui, X. Zhu, M. Liu, Y. Zhang, S. Yao. Fe-Single-Atom Nanozyme Catalysts for Sensitive and Selective Detection of Nitrite via Colorimetry and Test Strips. *ACS Appl. Nano Mater.*, 2023, **6**(7), 5879-5888.
[15] S. Wei, Y. Yang, J. Li, J. Wang, J. Tang, N. Wang, Z. Li. The Mn/Yb/Er triple-doped CeO_2 nanozyme with enhanced oxidase-like activity for highly sensitive ratiometric detection of nitrite. *Chin. Chem. Lett.*, 2024, **35**(6), 109114.
[16] Y. Xie, Y. Xu, H. Su, Z. Xie, Q. Kuang. $PtCu_3$ excavated rhombic dodecahedral nanocrystals with excellent peroxidase-like activity for highly sensitive colorimetric sensing of nitrite. *Food Chem.*, 2025, **485**, 144545.
[17] Y. Hao, Z. Wang, H. Wang, W. Dong, Y. Liu, Q. Hu, S. Shuang, C. Dong, Y. Guo, X. Gong. Rational design of carbon dot nanozymes for ratiometric dual-signal and smartphone-assisted visual detection of nitrite in food matrices. *J. Hazard. Mater.*, 2024, **480**, 136156.
[18] M. Wang, M. Zhao, P. Liu, H. Zhu, B. Liu, P. Hu, X. Niu. Coupling diazotization with oxidase-mimetic catalysis to realize dual-mode double-ratiometric colorimetric and electrochemical sensing of nitrite. *Sens. Actuators B Chem.*, 2022, **355**, 131308.
[19] J. Zhang, H. Chen, J. Liu, J. Gui, M. Liu, Y. Zhang, S. Yao. The target-induced redox and diazotized reaction for colorimetric ratio detection of nitrite using CoOOH nanosheets as mimetic oxidase. *Talanta*, 2023, **258**, 124458.
